# Evolutionary Constraints on Connectivity Patterns in the Mammalian Suprachiasmatic Nucleus

**DOI:** 10.3389/fnetp.2021.716883

**Published:** 2021-08-19

**Authors:** Connor Spencer, Elizabeth Tripp, Feng Fu, Scott Pauls

**Affiliations:** ^1^ Department of Mathematics, Dartmouth College, Hanover, NH, United States; ^2^ Department of Mathematics, Sacred Heart University, Fairfield, CT, United States; ^3^ Department of Biomedical Data Science, Geisel School of Medicine, Dartmouth College, Hanover, NH, United States

**Keywords:** evolutionary game theory, circadian rhythm, suprachiasmatic nucleus, connection topology, synchronization

## Abstract

The mammalian suprachiasmatic nucleus (SCN) comprises about 20,000 interconnected oscillatory neurons that create and maintain a robust circadian signal which matches to external light cues. Here, we use an evolutionary game theoretic framework to explore how evolutionary constraints can influence the synchronization of the system under various assumptions on the connection topology, contributing to the understanding of the structure of interneuron connectivity. Our basic model represents the SCN as a network of agents each with two properties—a phase and a flag that determines if it communicates with its neighbors or not. Communication comes at a cost to the agent, but synchronization of phases with its neighbors bears a benefit. Earlier work shows that when we have “all-to-all” connectivity, where every agent potentially communicates with every other agent, there is often a simple trade-off that leads to complete communication and synchronization of the system: the benefit must be greater than twice the cost. This trade-off for all-to-all connectivity gives us a baseline to compare to when looking at other topologies. Using simulations, we compare three plausible topologies to the all-to-all case, finding that convergence to synchronous dynamics occurs in all considered topologies under similar benefit and cost trade-offs. Consequently, sparser, less biologically costly topologies are reasonable evolutionary outcomes for organisms that develop a synchronizable oscillatory network. Our simulations also shed light on constraints imposed by the time scale on which we observe the SCN to arise in mammals. We find two conditions that allow for a synchronizable system to arise in relatively few generations. First, the benefits of connectivity must outweigh the cost of facilitating the connectivity in the network. Second, the game at the core of the model needs to be more cooperative than antagonistic games such as the Prisoner’s Dilemma. These results again imply that evolutionary pressure may have driven the system towards sparser topologies, as they are less costly to create and maintain. Last, our simulations indicate that models based on the mutualism game fare the best in uptake of communication and synchronization compared to more antagonistic games such as the Prisoner’s Dilemma.

## 1 Introduction

The mammalian suprachiasmatic nucleus (SCN) comprises about 20,000 oscillatory neurons networked through a variety of communication pathways that forms the master time-keeper for mammals (Mohawk et al., 2012). The SCN sits above the optic chiasm, receiving and incorporating optical input relaying the light/dark patterns of the outside world. These signals entrain the overall oscillation of the SCN to the external signal (Duffy and Czeisler, 2009), providing a robust circadian (circa = about, dian = day) rhythm that sets the timing for a myriad of biological processes (Ko and Takahashi, 2006).

The SCN has been the subject of intense study for decades where researchers have uncovered the molecular mechanisms for oscillation and many of the biological and chemical factors that influence a neuron’s oscillatory signal by speeding up or slowing down the rhythm (Ko and Takahashi, 2006). Further, oscillatory neurons must communicate through a network to facilitate construction and maintenance of the circadian signal (Welsh et al., 2010; Mohawk and Takahashi, 2011).

Interrogating the connectivity and communication structure for large collections of neurons is daunting. While experimentalists are always improving techniques, current technology comes with trade-offs that limit the scope of information we can collect. Consequently, while we know a great deal about the mechanisms of communication, we do not yet completely know how all of the communication fits together across the tissue.

Systems of coupled oscillators, such as the neurons in the SCN, have been modeled in numerous way to help better understand their function and properties. While many of these models contain detailed representations of biochemical processes, others are more abstract in modeling high level features of oscillatory systems (Asgari-Targhi and Klerman, 2019). Among the latter group of models, the Kuramoto coupled oscillator models are one of the most parsimonious and are ubiquitous in theoretical investigations of the SCN. In Kuramoto systems, as in the SCN (Welsh et al., 1995), if oscillators are disconnected completely from one another they revert to oscillating according to their intrinsic frequencies. But once connected, they can exhibit a wide range of partial or complete synchronization or none at all (Abrams and Strogatz, 2006; Zhou and Kurths, 2006; Allefeld et al., 2007; Hong and Strogatz, 2011; Ji et al., 2013; Pecora et al., 2014; Favaretto et al., 2017) depending on the patterns and strengths of the connections.

We see a basic evolutionary trade-off between the benefits of synchronization to the organism and the cost of the neural architecture it requires to facilitate the necessary communication. Synchronization creates a system-wide circadian rhythm that improves the survival (and hence reproduction) of the organism. Signals from the SCN regulate numerous functions across the organism including sleep schedules, metabolism, immune responses, activity levels, and hormone excretion (Lowrey and Takahashi, 2004). At the most basic level, a circadian rhythm allows the organism to enter energy saving modes at different times of day. At a more complex level, the timing and centralized clock that the SCN signal provides allows an organism the ability to predict the future—to know when it is safe to find food and avoid predation.

But what do we expect the communication and connectivity patterns to look like? To investigate this, we introduce a simple evolutionary game theoretic model built on a stripped down version of an oscillatory system. Earlier work by Tripp et al. (2020) derived analytical results for this model under the assumption that all oscillators were connected to all other oscillators. Here, we extend this investigation to test several different connection topologies. Arranging the oscillators on a lattice in the plane, we investigate the four and eight nearest neighbor topologies as well as randomly generated topologies with a mixture of nearby and long range connections.

Our simulations provide two central results. First, high levels of communication and connectivity are not necessary for a stable synchronous system: all of the topologies we tested produce fully communicative and synchronous systems in cost-benefit regimes similar to the all-to-all case. Second, the main difference between the topologies is the rate at which the systems converge to either full communication or non-communication. This has implications about the time scale of the potential evolution of synchronous systems—to arise in relatively few generations, the parameters in our model need to lie within a region where the underlying game in our evolutionary dynamics is mutualism. This type of game, also called harmony game (Hauert, 2001), is less antagonistic than other common model games such as the Prisoner’s Dilemma, as it has multiple positive outcomes. This is another useful feature as these outcomes correspond to phases of the oscillation, allowing our systems to synchronize to different phases.

## 2 Background and Related Literature

The SCN serves as the master circadian oscillator in mammals. Receiving input about external light cues from the optic nerve, the oscillatory neurons in the SCN synchronize to match the environment. This signal is disseminated throughout the body to other oscillatory centers, serving as a master time-keeper that allows the organism’s precise control over a wide variety of biochemical processes (Dibner et al., 2010). While oscillatory neurons in the SCN oscillate individually, interconnection and communication between them facilitates the synchronization that creates the master signal. Complex interactions along multiple channels of communication, including neurotransmitters and electrical firing, facilitate and regulate synchronization (Aton and Herzog, 2005).

The idea of using dynamic models to study properties of the SCN is not new. Researchers typically construct multicellular interaction models by first choosing a model of intra-cellular oscillation and then coupling these models together (see Asgari-Targhi and Klerman (2019) for a review). These models of cellular dynamics vary from relatively simple idealized models to much more complex models that reflect more of the biological detail. One of the most simple but effective models is the two-oscillator Kuramoto model (Acebrón et al., 2005), comprised of a pair of coupled ordinary differential equations which can be solved analytically. On the other end are frameworks such as that of Forger and Peskin (2003) (and its numerous adaptations and extensions), a system of 75 ordinary differential equations that model in detail many of the known processes involved in circadian oscillation within cells.

Many of the results from these types of models help us understand the role of different mechanisms in the SCN. For example, models that incorporate the neuropeptide vasoactive intestinal polypeptide (VIP) and/or the neurotransmitter *γ*-aminobutyric acid (GABA) demonstrate the impact of these signaling mechanisms on the circadian rhythms (To et al., 2007; Vasalou et al., 2009; DeWoskin et al., 2015; Myung et al., 2015). Similarly, other authors investigate the role of electrical signaling in the circadian system (Antle et al., 2003; Antle et al., 2007; Belle et al., 2009; Diekman and Forger, 2009; Noguchi et al., 2017).

Other investigations focus on the impact of connection patterns on the dynamics of the SCN, as we do in this paper but using other methodologies. Vasalou et al. (2009) use small-world connectivity (Watts and Strogatz, 1998) with a previous coupled differential equation dynamical model (To et al., 2007) modeling the effect of VIP on oscillations. With just 10% of the total possible connections, the small-world topologies produce phase synchronization across the model SCN. Bodenstein et al. (2012), inspired by experimentally observed seasonal differences in the dynamics of the SCN, use a local mean field coupling paired with random long range connections (similar to Vasalou et al. (2009)). They find that denser long-range connections lead to narrower phase distributions while sparse ones broaden the phase distribution, consistent with observed data in winter and spring respectively. Hafner et al. (2012) investigate the impact of random, local, and scale-free (Barabási and Bonabeau, 2003) topologies on SCN dynamics. Modeling the core and shell regions of the SCN, they find that scale-free connections in the core and local connections in the shell match experimental evidence most closely with the least number of connections. Gu et al. (2019) also consider connectivity between the core and shell, finding that the ratio of incoming and outgoing connections between the two impact the synchronization of the SCN during reentrainment. Šimonka et al. (2015) study the impact of stochasticity on oscillatory dynamics, finding that synchronization in models with small-world topologies is robust to even large stochastic perturbations.

Our work differs from those discussed above in that we incorporate aspects of evolutionary game theory in our model dynamics. In the most closely related work to ours, Antonioni and Cardillo (2017) integrate evolutionary game theory into the Kuramoto oscillator framework using a version of the Prisoner’s Dilemma to form the Evolutionary Kuramoto Dilemma (EKD). The primary features of the Kuramoto oscillator are its phase, denoted by *θ*_*i*_, its intrinsic frequency, *ω*_*i*_, and a global coupling strength, *λ*. The existence of coupling between the oscillators is encoded in an adjacency matrix, *H* = (*h*_*ij*_), where *h*_*ij*_ denotes the strength of the coupling from oscillator *j* to oscillator *i*. The simplest Kuramoto oscillator model (Kuramoto, 1975) is a system of coupled differential equations, one for each oscillator:

$${\theta ˙}_{i}=\omega_{i}+\lambda \overset{n}{\underset{j=1}{\sum }}h_{ij}\sin\left(\theta_{j}-\theta_{i}\right),$$ (1)

Antonioni and Cardillo overlay a strategy attribute onto each oscillator with two possibilities: *cooperation*, where neurons influence each other to move towards synchronization, and *defection*, where they don’t. This introduces additional parameters *s*_*i*_ ∈ {0, 1}, one for each oscillator, to the model

$${\theta ˙}_{i}=\omega_{i}+s_{i}\lambda \overset{n}{\underset{j=1}{\sum }}h_{ij}\sin\left(\theta_{j}-\theta_{i}\right),$$ (2)

where *s*_*i*_ = 1 signals cooperation and *s*_*i*_ = 0 yields defection. In the EGT framework, agents change their strategies by assessing a payoff function which, in the EKD model, is calculated by estimating solutions of the Kuramoto system (Eq. 1) and looking at oscillators’ level of synchronicity with their neighbors weighed against the cost of moving towards their neighbors average phase. The higher the net payoff, the more likely an agent will move to that strategy. This model allows the authors to investigate the co-evolution of synchronization and cooperative behavior. Among other findings they find a trade-off between the coupling strength and the cost in three types of connection topologies: Erdős-Rényi random networks (Erdős and Rényi, 1959), Barabási-Albert scale free networks (Barabási and Bonabeau, 2003), and random geometric graphs (Dall and Christensen, 2002). They find that cooperation and synchronization cannot occur at all unless the coupling strength rises above a threshold and that even for these higher coupling strengths, if the cost is too large synchronization and cooperation cannot persist.

As our work in this paper focuses on the impact of topology on synchronization and communicability, we review the work of several other authors who have extended the investigation of the Antonioni-Cardillo model by looking at the impact of other topologies on the dynamic processes. Yang et al. (2018) find that the synchronization level is highest when the underlying connection topology (given by the adjacency matrix *H* in Eq. 2) is of moderate average degree when using networks generated by the Erdős-Rényi random graph model (Erdős and Rényi, 1959). Liu et al. (2018) find that when the relative cost for cooperation is low, random topologies lead to synchronization and cooperation but as the cost rises, synchronization can only be achieved with the less random and more localized Watts-Strogatz small-world topologies (Watts and Strogatz, 1998). Zhou et al. (2019) find that, in some contrast to Yang et al. (2018), synchronization arises in data-derived networks with higher average degree and that strong synchronization is sufficient, but not necessary, for cooperation to arise. Further, looking at Erdős-Rényi and Barabási-Albert (Barabási and Bonabeau, 2003) networks they confirm that as average degree rises, synchronization becomes stronger.

## 3 Model Specification

We follow the method in Tripp et al. (2020) that introduced a different approach to studying synchronizable systems through an evolutionary game theoretic framework. Every evolutionary game theoretic model has several components: a collection of agents, strategies for each agent, a function which yields the payoffs that agents accrue when playing the game with one another, and a rule guiding the evolution of the strategies over time.

We define our agents as *n* oscillators with identical periods and assign them a pair of attributes, (*s*, *ϕ*_*i*_), that together give the agents’ strategies. The first attribute, *s*, is a flag that indicates whether an oscillator communicates with its neighbor, *s* = *C*, or is non-communicative, *s* = *N*. The second is the phase of the oscillator in its oscillation selected from {*ϕ*_1_, *…*, *ϕ*_*d*_}, where the *d* phases are distributed evenly around the circle, $\phi_{j}=j\frac{2\pi }{d}$ for *j* = 1, 2, *…*, *d*. As these provide a complete description of the oscillator, we denote oscillator *i* by the pair (*C*, *ϕ*_*i*_) if it is communicative and by (*N*, *ϕ*_*i*_) if it is not.

Our payoff functions depend on the flags and the distance between the phases on the circle. We have three basic assumptions that define our payoff:

1) An oscillator derives more benefit from the interaction the closer its phase is to its neighbors.
2) A communicative oscillator derives equal or more benefit than a non-communicative one as communication promotes synchronization which is beneficial to the organism.
3) Communication is Costly.

**Figure:**
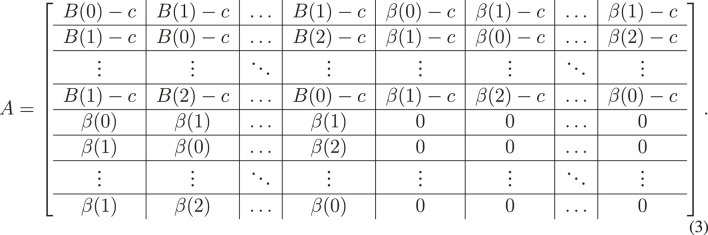


To codify this, we specify a cost associated to communication, *c*, and two benefit functions, *B* for oscillators that communicate and *β* for those that do not. The common domain of the benefit functions is the set $\left\{1,2,\ldots ,\left\lfloor \frac{d}{2}\right\rfloor \right\}$ , which represent the multiple of $\frac{2\pi }{d}$ given by the cyclic distance between two phases. To isolate this multiple, we first define the cyclic distance between phases:

$$\Delta \phi_{qr}=\left\{\begin{array}{cc} \left|\phi_{q}-\phi_{r}\right| & \text{if}\;\;\;\;\;\;|\phi_{q}-\phi_{r}|\leq \pi \\ 2\pi -|\phi_{q}-\phi_{r}| & \text{if}\;\;\;\;\;\;|\phi_{q}-\phi_{r}|>\pi \end{array}.\right.$$

To define the benefit function *B*, we isolate the multiple of $\frac{2\pi }{d}$ by letting

$$k=\left\{\begin{array}{cc} \left|q-r\right| & \text{if}\;\;\;\;\;\;|q-r|\leq \left\lfloor \frac{d}{2}\right\rfloor \\ d-|q-r| & \text{if}\;\;\;\;\;|q-r|>\left\lfloor \frac{d}{2}\right\rfloor \end{array}\right.$$

and defining *B*(*k*) to be *B*(Δ*ϕ*_*qr*_). The function *β* is defined analogously. To encode the first assumption above, we require both *B* and *β* to be non-increasing functions and to encode the second, we require that *B*(*k*) ≥ *β*(*k*) for all *k*.

We construct the payoff matrix *A* as a 2*d* × 2*d* matrix where both the rows and columns are indexed by the strategy list, $S=\left\{\left(C,\phi_{1}\right),\ldots ,\left(C,\phi_{d}\right),\left(N,\phi_{1}\right),\ldots ,\left(N,\phi_{d}\right)\right\}$ . Our convention is that *A*_*ij*_ gives the payoff to oscillator *i* when oscillators *i* and *j* interact. Taking advantage of these definitions and the symmetries of the phase differences, we have the payoff matrix given as

This matrix has four *d* × *d* blocks that guide what happens in four broad cases determined by the communication flag. The upper left-hand block gives payoffs for communicator-communicator interactions—the communicator pays a cost (*c*) but reaps a benefit *B*(⋅) depending on the similarity of the phases of the players. The upper right hand block gives payoffs for a communicator interacting with a non-communicator—they still pay the cost of communication but derive a potentially smaller benefit, *β*(⋅). The lower left hand block gives payoffs in the “free-rider” case, when a non-communicator interacts with a communicator. The payoff here is derived only from the function *β* as the non-communicator pays no communication cost. The last block, on the lower right, is the payoff for interaction between non-communicators. It is identically zero as the non-communicators neither gain nor lose anything due to the interaction. Within these blocks, the differences in the entries accounts for the differences between the phase portion of the strategies of the interacting oscillators. To use the matrix in calculating payoffs for oscillator *i*, playing strategy *S*(*i*), against oscillator *j*, playing strategy *S*(*j*), we define P(S(i),S(j)) to be the entry of the matrix with row corresponding to *S*(*i*) and column to *S*(*j*).

While the body of work related to the Antonioni and Cardillo’s EKD model described above considered the Kuramoto framework as a motivation, ours stems from studying oscillatory systems in the mammalian SCN. These biological systems are often modeled by coupled Kuramoto oscillators, but also provide descriptive features and constraints derived from biological experimentation. When oscillatory data is collected from SCN samples by recording movies of PER expression, the samples are first prepared by slicing them into relatively thin slabs. For this reason, we assume that the oscillators in our models are arranged on a plane. Second, studies of neural connectivity show that, typically, neurons most intensely communicate with other nearby neurons but also communicate with neurons further away, albeit more weakly (see the excellent review Bassett and Bullmore (2017)). Consequently, we focus on topologies with different mixtures of local and long-range connectivity within this broad description. Here, we recognize a limitation of our approach in that our framework does not allow for varying the intensity of the interaction.

The prior studies on the properties of the EKD model, while enlightening, invariably assume the Prisoner’s Dilemma type interactions among oscillators, specifically in the form of a donation game. Our setup is more flexible as many more classical games can arise in addition to the Prisoner’s Dilemma depending on the choices of *B*(0), *β*(0) and *c*, as shown in Figure 2.

## 4 Methods

Our goal is to use the evolutionary game theoretic model we introduced above to better understand under what conditions communication among the oscillators can arise. Our basic setup is to create a collection of oscillators with the same phase that do not communicate with one another. Then, we introduce a single oscillator that does communicate and has a phase that is maximally different from the phases of the other oscillators. Introducing a single new element mimics a mutation enabling communication between oscillators arising in the system. The choice of initial phases is set to make the adoption of the communicative strategies as difficult as possible.

With these initial constructions, we then apply a death-birth process (Ohtsuki et al., 2006), a common update process used in evolutionary game theory to mimic evolution within the system, to see the impact of the invasion of the communicative strategy. If we were to run this process infinitely many times, it will reach an equilibrium of either all communicative oscillators or all non-communicative ones. In this work, we use a fixed finite number of iterations. At the end of the simulations, we examine the proportion of communicative oscillators across different parameters in the model to quantify the success of the invasion.

To formalize this, we need to describe several details of the model: the payoff functions, *B* and *β*; the cost, *c*; the death-birth process; and the connectivity pattern of the oscillators.

### 4.1 Payoff Functions

The model introduced in Tripp et al. (2020) has two baseline assumptions about *B* and *β*: both are monotonic non-increasing functions, and *B*(*k*) ≥ *β*(*k*). For this work, we choose both to be linear functions:

$$B\left(\Delta \phi \right)=B\left(0\right)\left(1-\frac{\Delta \phi }{d}\right),\;\;\beta \left(\Delta \phi \right)=\beta \left(0\right)\left(1-\frac{\Delta \phi }{d}\right),$$

where *d* is the number of possible phases for the oscillators and *B*(0) ≥ *β*(0). As in Tripp et al. (2020), we will examine a range of values for *B*(0) and *β*(0) to see when communication is favored. We define the cost of communication, *c*, in one of two ways, depending on the connection topology of the oscillators. For cases when every oscillator has the same number of connections as every other oscillator, we fix a global cost of *c* = 10. For cases where oscillators have different numbers of connections, the cost to oscillator *i* is $c\left(i\right)=10\frac{N\left(i\right)}{\mu }$ where *N*(*i*) is the number of other oscillators that *i* is connected to and $\mu =\frac{1}{n}\sum_{j=1}^{n}N\left(j\right)$ , the mean number of connections per oscillator. This definition of *c*(*i*) ensures that the mean cost, $c¯=\frac{1}{n}\sum_{j=1}^{n}c\left(j\right)$ , is equal to 10, ensuring that simulations with this cost structure are comparable to those with the global fixed cost of 10 (Masuda, 2007).

Our choice of this cost structure stems from practical computational considerations. In exploring the parameter space, we focus on understanding the impact of *B*(0) and *β*(0) values that are both above and below different multiples of the cost. Setting the cost (or the expected cost) at 10 allows us to complete simulations for *B*(0) and *β*(0) up to values of 40 in a reasonable amount of time, and with sufficient resolution between multiples of *c* to draw conclusions about how simulations behave in these scenarios.

### 4.2 The Death-Birth Process

Strategies in our network of oscillators change according to a death-birth process. The outline is simple: for each iteration, we pick an oscillator uniformly at random to remove from the system and replace it with a new oscillator whose strategy is determined by the potential payoff of different strategies for that node. To make this precise, we need to quantify the payoffs for given strategies, which requires a few definitions. If (*i*, *S*(*i*)) denotes an oscillator *i* with strategy *S*(*i*), we first define the *expected payoff*, denoted by *π*_(*i*,*S*(*i*))_, as the average payoff received if *i* plays its strategy with its neighbors according to the payoff matrix (**Eq. 3**):

$$\pi_{\left(i,S\left(i\right)\right)}=\overset{n}{\underset{j=1}{\sum }}M_{ij}P\left(\left(i,S\left(i\right)\right),\left(j,S\left(j\right)\right)\right)$$ (4)

here, *M*_*ij*_ indicates the connectivity between oscillators *i* and *j*:

$$M_{ij}=\left\{\begin{array}{c} 0\;\text{ if oscillators }i\text{ and }j\text{ are not connected,}\;\\ 1\;\text{ if oscillators }i\text{ and }j\text{ are connected.}\; \end{array}\right.$$ (5)

Finally, we can define the *fitness* of an oscillator *i* with strategy *S*(*i*) as $f_{\left(i,S\left(i\right)\right)}=e^{\delta \pi_{\left(i,S\left(i\right)\right)}}$ , where the parameter *δ* is the strength of selection (following the framework in Traulsen et al. (2008)). In this work, we always take *δ* = 1.

With these preliminary definitions in place, we can describe the selection of a strategy in the birth stage of the death-birth process. If the random draw selects node *i*, we compute the *fitness profile* for node *i*, the vector of fitnesses for node *i* adopting one of its neighbors’, say *j*’s, strategy, normalized to be a probability distribution:

$$F_{j}={\left(\frac{f_{\left(j,S\left(j\right)\right)}}{\sum_{k}f_{\left(k,S\left(k\right)\right)}}\right)}_{k\in N_{i}},$$

with $N_{i}=\left\{k|M_{ik}=1,k=1,\ldots ,n\right\}$ to denote the set of nodes of *i*’s direct neighbors. We then select a strategy for the new oscillator randomly according to this probability distribution.

Given the complexity of these definitions, we want to emphasize several properties. First, this construction ensures that it is more likely for the new node to have a high fitness strategy, but the randomness allows for sub-optimal strategy assignments. Second, the fitness profile for different oscillators may be very different from one another if the two oscillators are connected to different collections of other oscillators. Third, in contrast to the last point, if two oscillators are connected to the same set of other oscillators, their fitness profiles will be the same.

### 4.3 Connection Topologies

In calculating the payoffs that guide the death-birth process, we need to specify which other oscillators a specific oscillator has the possibility of interacting with. We do this by defining a connection topology encoded in an *n* × *n* matrix *M* given as in (Eq. 5).

**FIGURE 1:**
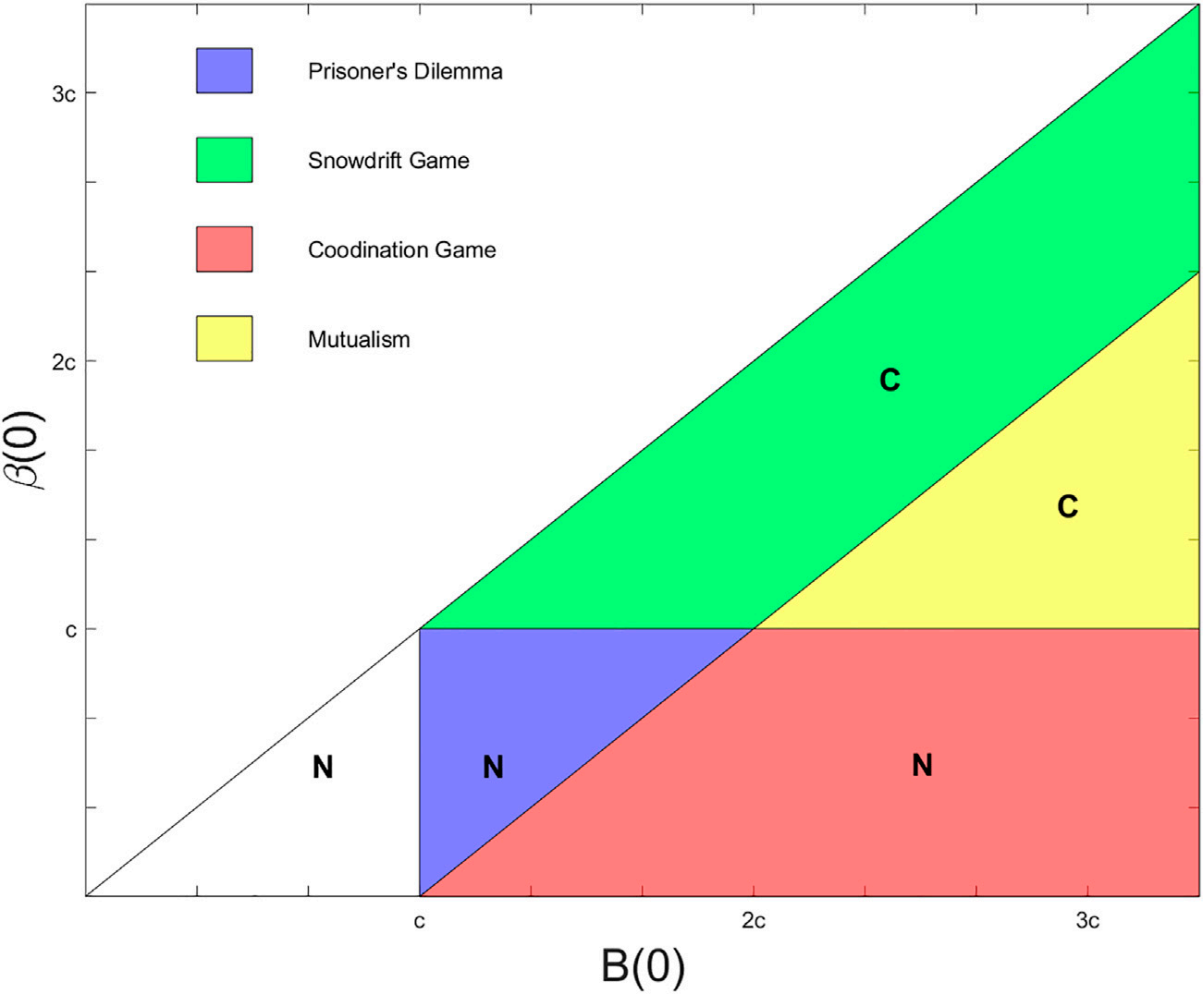
Spatial topologies of oscillator interactions. A schematic of the arrangement of 100 oscillators along an integer grid in the two-dimensional plane. Three nodes highlighted with a colored outline in blue (also labeled (2,2)), purple ((9,9)), and red ((3,6)) give examples of 4 nearest neighbor (von Neumann neighborhood), 8 nearest neighbor (Moore neighborhood), and probabilistic spatial topologies respectively.

Tripp et al. (2020) focused on the *all-to-all* topology, where every oscillator is connected to every other oscillator. We can represent this by a matrix *M* where *M*_*ij*_ = 1 for all distinct pairs of *i* and *j*. Using the all-to-all topology as a point of comparison, we will examine three additional biologically plausible topologies, all of which are variants of nearest-neighbor topologies. To describe these, we begin by arranging the oscillators on a 2-dimensional integer grid labeled using a coordinate pair (*x*, *y*). Figure 1 shows the arrangement and labeling with 100 oscillators.

The 4 nearest neighbor topology connects an oscillator at location (*x*, *y*) with the others at locations (*x* + 1, *y*), (*x* − 1, *y*), (*x*, *y* + 1), and (*x*, *y* − 1) if those locations are defined; if an oscillator is located on the edge of the grid, it omits whichever connections cannot be formed. The 8 nearest neighbor topology expands on the connections in the 4 nearest neighbor case by adding connections to oscillators at (*x* + 1, *y* + 1), (*x* + 1, *y* − 1), (*x* − 1, *y* + 1), and (*x* − 1, *y* − 1), if oscillators exist at those locations. The probabilistic spatial topology introduces randomness into the definition of connections, creating the possibility that different oscillators will have different numbers of connections. In this case, we place an edge between distinct oscillators labeled (*x*_1_, *y*_1_) and (*x*_2_, *y*_2_) with probability

$$p=\frac{1}{\sqrt{{\left(x_{2}-x_{1}\right)}^{2}+{\left(y_{2}-y_{1}\right)}^{2}}+\gamma },$$

where *γ* is a constant parameter with which we can manipulate the density of the connections: smaller values of *γ* create denser patterns while larger ones create sparser ones. Given a fixed *γ*, this construction makes connections between nearby oscillators more likely than ones that are far away as $\sqrt{{\left(x_{2}-x_{1}\right)}^{2}+{\left(y_{2}-y_{1}\right)}^{2}}$ gives the Euclidean distance between the grid points where the oscillators are placed.

The probabilistic spatial topology has commonalities with the Watts-Strogatz small-world topology. While both have a mixture of nearby and long-range connections, the generative mechanisms are different. Typically, small-world networks start with a fixed nearest-neighbor topology (like one of the ones above) and add a certain proportion of long-range connections at random. In the probabilistic spatial topology model all connections arise with probability dictated by the distance between the two oscillators on the plane. Consequently, there are fewer connections that are very long and more when distances are small. This leads to cases where we would not classify networks with the probabilistic spatial topology as small world. For example, with *γ* = 10 the average clustering coefficient over 100 instances is approximately 0.0365 while the average path length is 2.08.

### 4.4 Simulations

For our simulations, we fix the various parameters as shown in Table 1 and execute the death-birth process for 50,000 iterations. To construct our topologies, we arrange the 1,600 oscillators in a 40 × 40 integer grid and use the processes described above. For the 4 and 8 nearest neighbor topologies, as well as the all-to-all topology, we use a fixed uniform cost *c* = 10. For the probabilistic spatial topology, we use the normalization to enforce an average cost of 10.

**TABLE 1:** Parameters that we use in simulations of our evolutionary game theoretic model.

Description	Parameter	Value(s)
Payoff benefit (communicators)	*B*(0)	{1, *…*, 40}
Payoff benefit (non-communicators)	*β*(0)	{1, *…*, *B*(0)}
Payoff cost	*c*	10, or degree adjusted
Number of oscillators	*n*	1,600
Number of phases	*d*	10
Selection strength	*δ*	1
Number of iterations		50,000

To measure the evolution of the strategies of the oscillators, for each iteration we record the percentage of oscillators with a communicative strategy, *p*. Consequently, the vector of these probabilities, $\vec{p}$ , shows not only if the system converges to an all communicative or all non-communicative state, detected by a 1 or 0 in the last entry of $\vec{p}$ , but also how long and what path the system took to get there. While some evolutionary game theoretic frameworks introduce a component of random mutation, we do not and consequently, once *p* reaches 0 or 1, the probabilities cannot change again no matter how many additional iterations we perform. For simulations where *p* does not settle on either 0 or 1, we end the simulation after 50,000 iterations of the death-birth process, and calculate the average percentage of the communicative strategy in a mixed state, with some communicative and some non-communicative oscillators. This simulation procedure is in line with prior common practice for games on graphs (Santos and Pacheco, 2005).

## 5 Results

**FIGURE 2:**
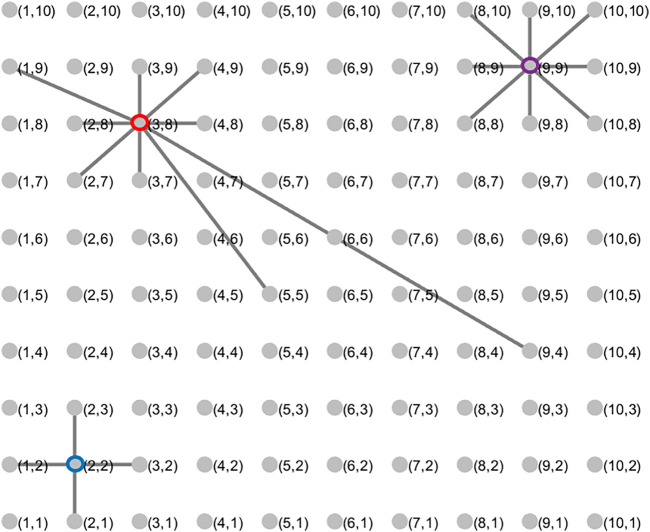
Beyond the Prisoner’s Dilemma. For different values of *B*(0) and *β*(0), our evolutionary game theoretical model encompasses four regimes of classical games: Prisoner’s Dilemma (blue), Snowdrift (green), Coordination (red), and mutualism (yellow). The regions are marked if we expect a communicative invasion of a non-communicative population to take over (C) or fail (N) with all-to-all connectivity. Results from Tripp et al. (2020).

To contextualize the simulation results for the different topologies, we recall some of the theoretical results in Tripp et al. (2020). Tripp et al. which show that different choices of *B*(0) and *β*(0) can produce one of four different games—the Prisoner’s Dilemma, the snowdrift game, the coordination game, and mutualism, which are shown as the blue, green, red, and yellow regions in Figure 2 respectively. When considering the all-to-all topology, the authors find conditions for the success of a strategy invading a population with strategies of the other communicative type. For the set-up described in the previous section, where a single communicative oscillator invades a pool of non-communicative ones, those results translate to the labeling of the regions in Figure 2. In short, so long as *B*(0) ≥ *β*(0) > *c*, we expect the invasion to be successful.

**FIGURE 3:**
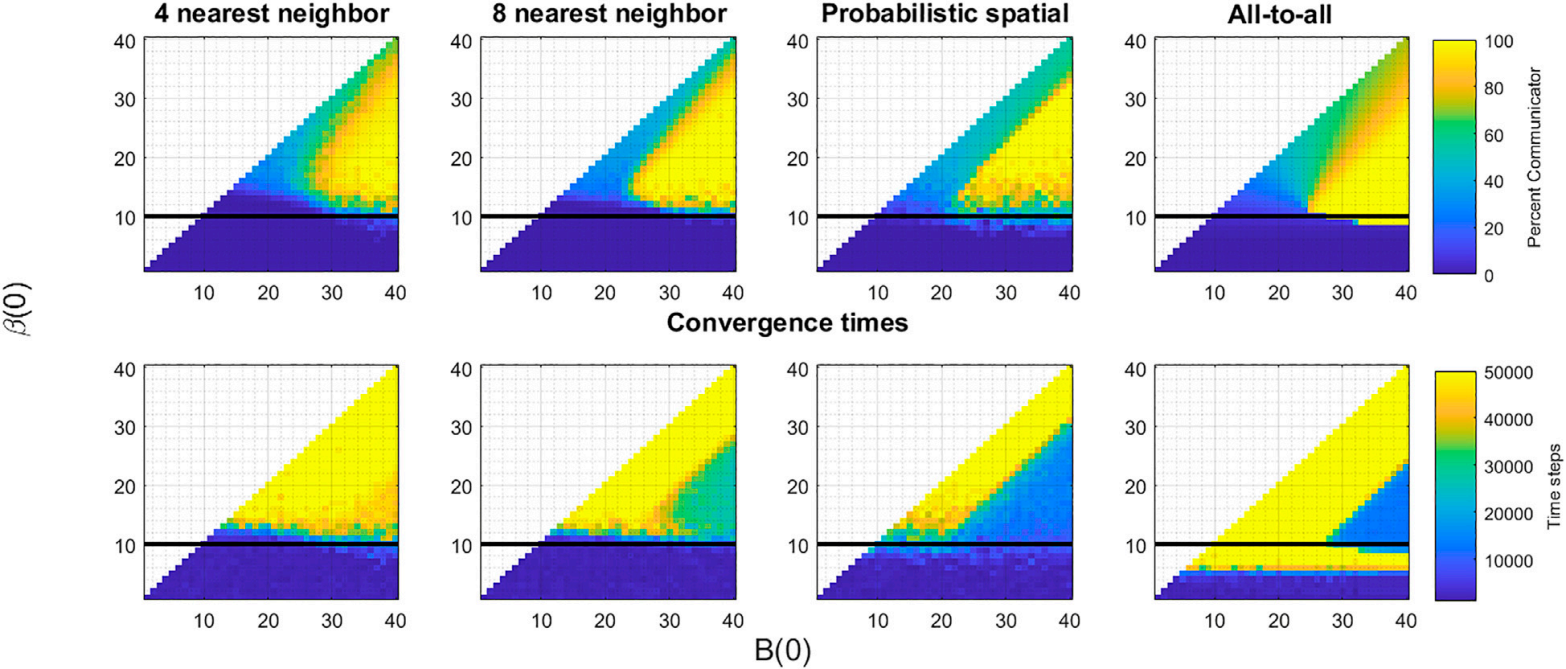
Impact of different topologies. For each of the four topologies—four, eight, and probabilistic spatial topology, and all-to-all—we show the results of the introduction of a single communicative oscillator after 50,000 iterations of the death-birth process **(top row)** and the convergence time **(bottom row)**. On each of the panels, the dark line is placed at the cost (or average cost in the probabilistic spatial topology case) indicating the theoretical boundary shown in Figure 2 for the all-to-all topology for comparison. Broadly, we see that as connectivity becomes more dense, convergence times drop and more communicative invasions are successful. But, particularly in the mutualism game region, all topologies result in cases of full communication and hence synchronization.

Generally, our simulation results are consistent with Tripp et al.’s analytic results. Discrepancies are likely due to the changes in topology, the finite number of iterations of the process, or both. Figure 3 shows the results of the simulations described in the previous section for four topologies—the 4 and 8 nearest neighbor topologies, the probabilistic spatial topology with average degree 10, and the all-to-all topology. In the top row, the color of the pixel corresponding to a (*B*(0), *β*(0)) pair shows the percent of communicative oscillators after 50,000 iterations, averaged over twenty trials. In the bottom row, a similar pixel shows the average over the trials of the number of iterations it takes the system to converge to either all communication or all non-communication. A value of 50,000 indicates that none of the twenty trials converged to a uniform strategy by the end of the experiments. Each of the panels on the top row are broadly similar, having the same three features: a triangular region where the systems converge to full communication, a parallelogram above the first region where the systems reach 25–80% communication by the end of the iterations, and another (rough) parallelogram at the bottom of the images where the systems converge to full non-communication. Comparing to Figure 2, we see the first region corresponds to the mutualism game, the second to the snowdrift region, and the third to the union of the coordination and Prisoner’s Dilemma region. Beyond these similarities, we see the trend that higher degree corresponds to better delineation of these regions.

The results shown in the bottom row further reveal that higher degree corresponds to faster convergence and that introducing some long range connections in the probabilistic spatial topology speeds convergence up even more. This is particularly evident in the triangular region associated with the mutualism game. In the four nearest neighbor topology, many of the (*B*(0), *β*(0)) pairs do not reach a uniform strategy by the end of 50,000 iterations. But as we move through topologies with higher degrees, convergence times drop quickly. The change from the eight nearest neighbor topology to the probabilistic spatial topology is most stark. Moving from a uniform degree of eight to an average degree of ten cut the convergence time roughly in half, reinforcing the impact of the randomization and longer range connections introduced by the probabilistic framework. The snowdrift region is very slow to converge across all choices of topology, which is consistent with theoretical results showing convergence in snowdrift games can be exponential in time (Antal and Scheuring, 2006). The regions corresponding to Prisoner’s Dilemma and coordination games show very fast convergence to a uniform population of non-communicators—the invasion by the communicative strategy fails.

## 6 Discussion

Our first observation drawn from the results shown in Figure 2 is that high levels of communication and connectivity are not necessary to achieve a stable synchronous system. While the sparser topologies converge more slowly than denser ones, the results are qualitatively the same—the regions where communicative strategies or non-communicative strategies become dominant are similar across the topologies we explored. The differences are indicative of the differences in convergence times. For example, in the all-to-all topology, the region in the lower right hand corner of Figure 3A has a sharp delineation between the region where the system is fully communicative (yellow) or fully non-communicative (blue). The same region in the nearest-neighbor topologies is less well separated, with a transitional region between the yellow and blue regions where there are mixtures of communicative and non-communicative oscillators. As the death-birth process that we defined at the foundation of the model is a Markov chain with absorbing states that are either fully communicative or fully non-communicative, these systems would eventually have the same sharp division if we completed (many) more iterations. This is consistent with the observed connectivity in neural systems, which are densely connected locally but much more sparsely connected at long ranges, and our model provides a potential explanation for this configuration. Among the many processes and constraints that impact the evolution of a species, two important factors stand out when considering a circadian time-keeper—the benefit of minimizing the biological cost of the system and the benefit the system confers on the organism in terms of its survival. In our exploration, these are in tension—the benefit of synchronization drives the system to enable more communication, but this comes at a cost in the biological construction and maintenance of the system. Our results support the hypothesis that the relatively sparse topology in the SCN that can still achieve synchronization is a consequence of optimizing these forces.

Second, we observe that on evolutionary times scales, in most cases we should expect a mixture of communicative and non-communicative oscillators. Our simulations ran for the equivalent of 50,000 generations—a very long time period for most mammals. Our simulation results show that for many combinations of *B* and *β*, our models do not converge to complete communication or complete non-communication within this time frame. Consequently, in organisms that developed an SCN-type circadian clock within a relatively recent evolutionary time scale, we should expect to see a combination of communicative and non-communicative oscillators. But, this interpretation is confounded by some, not completely known, features of the evolutionary landscape. Circadian clocks are almost ubiquitous across all organisms, although the mechanisms differ. On one hand, this suggests that either clocks emerged very early and continued to be refined through evolution and speciation. On the other, external selection pressure for some sort of time keeper may create the conditions for convergent evolution of similar clock mechanisms in different species, arising more recently and independently. As we do not have complete knowledge of the progression, this interpretation of our results is tempered.

With this caveat in mind, the results of the simulations lead us to a conjecture about the factors that constrain the development of a system such as the SCN. Considering the desirable and observed properties of synchronous systems leads us to identify several key components. First, the evolutionary framework should lead to a communicative, hence synchronous, system given the ubiquity of biological mechanisms like the SCN across species. Second, the convergence to a uniform communicative system should be reasonably fast to be plausible on an evolutionary time scale. Third, once a system reaches a fully communicative state, it should be stable and resistant to perturbations that disrupt communication. Fourth, the system should be flexible in that it can converge to any phase within the collection of phases. And last, the connectivity of the system should be as sparse as possible while achieving the other components to minimize biological cost. Of our four regions, only mutualism demonstrates all of these properties. Pairs in that region converge to full communication, unlike those within the coordination game and the Prisoner’s Dilemma regions. Unlike the snowdrift region, convergence is fast with low average degree when using the probabilistic spatial topology. Like the coordination game but unlike snowdrift and the Prisoner’s Dilemma, mutualism is agnostic to the question of the phase to which it converges. Hence, the resulting systems retain the ability to change phases. Last, as the cost is smaller than either *B*(0) or *β*(0) in the mutualism region, once the systems have reached convergence, they are difficult to move.

Taken together, these results both complement and extend the work on Antonioni and Cardillo’s Evolutionary Kuramoto Dilemma model (Antonioni and Cardillo, 2017). While the frameworks are different from one another, some of the results point in the same direction—the systems converge when the topologies have reasonably moderate degrees and topologies akin to Watts-Strogatz small-world models facilitate better convergence properties. Our results refine these further, delineating cases where topologies can be very sparse and yet still enable reasonably fast convergence to communication and synchronization. Moreover, our identification of the mutualism game as the most plausible model within our framework stands in contrast to the use of the Prisoner’s Dilemma as a model for pairwise interactions in the earlier work (Antonioni and Cardillo, 2017; Liu et al., 2018; Yang et al., 2018; Zhou et al., 2019), as well as models where oscillators change their connectivity to maximize their influence within the system (Brede et al., 2018). While interactions in these other models produce an antagonistic environment where the collective’s best action is at odds with an individual’s rational action, mutualism is more harmonious, as players of the game are motivated to pick one of the multiple strategies that is best for the whole (Hauert, 2001). This is reflective of the differing goals of the models. The other models cited are designed to explore the rise of synchronization in coupled oscillator systems in the face of adverse conditions imposed by costly connectivity. Our current model has similarities, but more importantly, it takes into account all possible types of pairwise game theoretic interactions that can arise in neuronal populations, including but not limited to the Prisoner’s Dilemma games. In sum, the present work is further motivated by the development of synchronous oscillatory systems in organisms from the perspective of evolutionary dynamics that are driven by the exquisite tradeoff of benefit and cost of communication. The ubiquity of these systems implies that the evolutionary benefits substantially outweigh the costs, making a more relaxed model such as mutualism reasonable.

## Data Availability

The raw data supporting the conclusion of this article will be made available by the authors, without undue reservation.
